# Magnitude and determinants of breastfeeding initiation within one hour among reproductive women in Sub-Saharan Africa; evidence from demographic and health survey data: a multilevel study

**DOI:** 10.1186/s12889-022-13114-y

**Published:** 2022-05-28

**Authors:** Tilahun Yemanu Birhan, Muluneh Alene, Wullo Sisay Seretew, Asefa Adimasu Taddese

**Affiliations:** 1 Department of Epidemiology and Biostatistics, Institute of Public Health, College of Medicine and Health Science, University of Gondar, Gondar, Ethiopia; 2 Department of Public Health, College of Health Science, Debre Markos University, Debre Markos, Ethiopia

**Keywords:** Early initiation of breastfeeding, Optimal breastfeeding, Multilevel, And Sub-Saharan Africa

## Abstract

**Background:**

Early initiation of breastfeeding is one of the most simple and essential intervention for child development and survival in the world. World Health Organization recommended to begin breast milk with one hour after delivery. The objective of this study was to determine the magnitude of early initiation of breastfeeding in Sub-Saharan Africa using DHS data set.

**Methods:**

This study was carried out within 32 Sub-Saharan African countries from 2010–2020, a pooled study of early initiation of breastfeeding was performed. For assessing model fitness and contrast, intra-class correlation coefficient, median odds ratio, proportional change in variance, and deviance were used. In order to identify possible covariates associated with early initiation of breastfeeding in the study area, the multilevel multivariable logistic regression model was adapted. Adjusted Odds Ratio was used with 95% confidence interval to declare major breastfeeding factors.

**Result:**

The pooled prevalence of early initiation of breastfeeding in Sub-Saharan Africa countries was 57% (95% CI; 56%—61%), the highest prevalence rate of early initiation of breastfeeding was found in Malawi while the lowest prevalence was found in Congo Brazzaville (24%). In multilevel multivariable logistic regression model; wealth index (AOR = 1.20; 95% CI 1.16 – 1.26), place of delivery (AOR = 1.97; 95% CI 1.89 – 2.05), skin-to-skin contact (AOR = 1.51; 95% CI 1.47 – 1.57), mode of delivery (AOR = 0.27; 95% CI 0.25 – 0.29), media exposure (AOR = 1.36; 95% CI 1.31 – 1.41) were significantly correlated with early initiation of breastfeeding in Sub-Saharan Africa.

**Conclusion:**

The magnitude of early initiation of breastfeeding rate was low in Sub-Saharan Africa. Covariates significantly associated with early initiation of breastfeeding was wealth index, place of delivery, mode of delivery, women educational status, and media exposure. Structural improvements are required for women with caesarean births to achieve optimal breastfeeding practice in Sub-Saharan Africa.

## Introduction

Breastfeeding is a universally acceptable essential nutrient that protects children from infectious and chronic illness overall the world [1, 2]. Globally, more than 60% of infant and young child deaths happens due to inappropriate infant feeding practice [1]. Early initiation of breastfeeding (EIBF) is one of the most simple and essential intervention for child development and survival in the world. World Health Organization recommended to begin breast milk with one hour after delivery [3, 4]. Early initiation of breastfeeding has the ability to prevent 22% of neonatal deaths if all infants were breastfed within an hour after delivery [5, 6]. EIBF has thoughtful implication for both infants and mothers regarding of nutritional, developmental and immunological outcome [7, 8]. The practice of EIBF enables further provision of immunoglobulin and other vital bioactive molecule-rich colostrum for newborns that are critical for their immunity, growth and development [4, 9]. In addition, EIBF practice encourages bonding between child and mother resulting in legitimate outcome for infant and child development [10–12]. Further, EIBF practice has an implication for both short and long-term benefit for mothers in the case of reducing postpartum haemorrhage, lower risk of obesity in post-delivery, advance in birth spacing period, as well as reduces the risk of breast and ovarian cancer in the long run [13, 14]. The global public health recommendation indicates that infants should be exclusively breastfed for the first six months extending up to 24 months with additional foods [15]. Evidences of early breastfeeding initiation suggests that, timely and exclusive breastfeeding is one of the most top effective intervention to improve child health and growth [2, 16–19]. Evidence suggests that early initiation of breastfeeding has the ability to prevent 823, 00 annual deaths among under five children and it prevents 20, 000 annual deaths from breast cancer [1]. Despite the necessities of EIBF, delayed initiation of breastfeeding and prelacteal feeding are highly practiced in low and middle income countries resulting in a considerable increase in infant mortality and overall disease burden [5, 20–22]. Hence the magnitude of delayed initiation and practice of prelacteal feeding was high in resource limited countries like Sub-Saharan Africa since provision of health care as well as accessing health service are poor in this area [6, 15, 18, 23]. Also, the practice of prelacteal feeding is considered as normal nutritional benefit like breast milk and supported by traditional birth attendants and priests, this are one the most obstacle to promote early initiation of breastfeeding and to maximize optimal breastfeeding in this area [14, 24, 25]. Hence low rate of timely breastfeeding initiation is one of the major global health problems, which is the contributing factor for childhood undernutrition, morbidity, mortality, impaired intellectual development, suboptimal adult work capacity, and increased the risk of in the adulthood [5, 7, 24]. Previously published reports suggested that infant feeding behaviour including timely initiation of breastfeeding play in important role in reducing child morbidity and mortality [15, 16, 18, 21, 24, 26, 27]. However, there is no studies investigated pooled prevalence of early initiation of breastfeeding in Sub-Saharan Africa especially using the standard DHS data. This study aimed to determine the pooled prevalence of early initiation of breastfeeding in Sub-Saharan Africa using DHS data set. The finding of this study will give relevant information to international communities to assess the scope of optimal breastfeeding and for further targeted intervention in Sub-Saharan African countries.

## Method

### Source of data

The data was obtained from the measure DHS program at www.measuredhs.com after prepared concept notes about the project. The demographic and Health Survey (DHS) data were pooled from the 32 Sub-Saharan Africa (SSA) countries from 2010 to 2020. The Sub-Saharan African continent consists of 54 recognized countries. Geographically, sub-Saharan Africa is a region situated south of the Sahara desert on the continent of Africa. Sub-Saharan Africa, according to the United Nations (UN), consists of all African countries which are entirely or partially located south of the Sahara. As part of Sub-Saharan Africa, the UN Development Program recognizes 46 out of 54 African countries, while the World Bank mentions Somalia and Sudan. The recent DHS of country specific dataset was extracted during the specified period.

In this study, 34 countries in the sub region met our selection criteria (sub-Saharan African countries that possessed DHS data sets between 2010 and 2020) available in the public domain. The countries were Angola, Benin, Burkina Faso, Burundi, Cameroon, Cote d’Ivoire, Comoros, Congo Brazzaville, Democratic Republic of Congo, Ethiopia, Gabon, Gambia, Ghana, Guinea, Kenya, Lesotho, Liberia, Malawi, Mali, Namibia, Niger, Nigeria, Rwanda, Senegal, Sierra Leone, Tanzania, Uganda, Zambia, and Zimbabwe.

The DHS program adopts standardized method involving uniform questionnaires, manuals, and field procedures to gather the information that is comparable across countries in the world. DHSs are nationally representative household surveys that provide data from a wide range of monitoring and impact evaluation indicators in the area of population, health, and nutrition with face to face interviews of women age 15 to 49. The surveys employ a stratified, multi-stage, random sampling design. Information was obtained from eligible women aged from 15 to 49 years in each country. The detailed methodology of the survey and the process used to collect the data have been recorded elsewhere [28].

### Variables

#### Outcome variable

The outcome variable, early/timely initiation of breastfeeding, was determined by asking mothers for details about when their babies were placed on their breasts after birth. The ratio of children placed to the breast within one hour of birth to the total number of children was used to calculate the prevalence of early breastfeeding initiation.

### Independent variables

Variables in socio-demographics and the economy (residence, region, maternal age, marital status, religion, maternal education, paternal education, wealth index, maternal occupation/maternal working Status), Pregnancy and factors linked to pregnancy ( ANC visit, Parity, Preceding birth interval, contraceptive use, Place of delivery, Birth order, Mode of delivery, size of child at birth). Behavioural factors.

(Smoking, media exposure) were included for this study.

#### Community-level variables

Non-aggregate community-level variables were place of residence and area. The place of residence has been registered as rural and urban. The area was described as the province from which a child comes from. By aggregation from an individual level, another group of community-level variables was developed using average approaches to conceptualize the neighbourhood effect on the implementation of EIBF. Education for women in the neighbourhood, community poverty, community visit to the ANC, community place of delivery.

### Data management and analysis

The research for this thesis was performed using version 15 of STATA (STATA Corporation. IC., TX, USA). For the calculation of descriptive statistics such as proportions, sampling weights were used to account for non-proportional distribution of the sample to strata. In the case of standard regression models, the research participants are considered to be independent of the outcome variable. Nevertheless, units in the same category are rarely independent when data is ordered in hierarchies [29]. Units from the same setting (cluster) are more similar to each other in relation to other units, or in relation to the outcome of interest, than units from another setting. This may then lead to a breach of the assumption of independence which could have the effect of underestimating standard errors and increasing Type I error rates (increases rate of false positivity of our results). In such circumstances, multilevel modelling can simultaneously account for person and community-level variables and provide a more comprehensive understanding of early initiation of breastfeeding factors [30].

### Multi-level analysis

Multilevel models are therefore developed to overcome the analytical problems that arise when data is hierarchically organized, and sampled data is a sample of several stages of this hierarchical population, such as DHS, in which children are nested in households, and households are nested in clusters, and there is an intra-group correlation. In order to estimate both independent (fixed) effects of explanatory variables and community-level random effects on the initiation of prelacteal feeding, a two-level mixed-effect logistic regression model was fitted. The person (children) is the first level and the cluster is the second level (community). In the bi-variable multilevel logistic regression model, the individual and community level variables associated with early initiation of breast feeding were independently tested and variables that were statistically significant at *p*-value 0.20 were considered for the final individual and community level adjustments. In the multivariable multilevel analysis, variables with *p*-value < 0.05 were declared as significant determinants of early initiation of breast feeding.

Therefore, using the two-level multilevel model, the record of the likelihood of implementing prelacteal feeding was modelled as follows:

$$\mathrm{log}\left(\frac{{\pi }_{ij}}{1-{\pi }_{ij}}\right)={\beta }_{0}+{\beta }_{1}{X}_{ij}+{\beta }_{2}{Z}_{ij}+{\mu }_{j}$$

where, i and j are the units of level 1 (individual) and level 2 (population) respectively; X and Z apply to variables of the individual and community level, respectively; ${\pi }_{ij}$ is the likelihood of having prelacteal feeds in the j^th^ community for the i^th^ mother; the β's are the fixed coefficients-therefore, there is a corresponding efficiency for each one-unit increase in X/Z (a set of predictor variables). Whereas, in the absence of control of predictors, ${\beta }_{0}$ is the intercept-the effect on the likelihood of mother on the provision of prelacteal feed; and μ_j_ indicates the random effect for the j^th^ community (effect of the community on the decision of mother to provide prelacteal feed). The clustered data existence and the within and between community variations were taken into account by assuming that each community has a different intercept (${\beta }_{0}$) and fixed coefficient (β).

### Model building

A total of four models were fitted. The first was a null model with no exposure variables, which was used to determine random effects at the population level and assess for heterogeneity in the community. Then model I was the multivariable model adjustment for individual-level variables and model II which was adjusted for community-level factors. In model III, the outcome variable was equipped with potential candidate variables from both person and community-level variables.

### Parameter estimation method

Fixed effects (an association measure) were used to estimate the relationship between the likelihoods of EIBF and explanatory variables at both the population and person level, and the results were expressed as odds ratios with a 95% confidence interval. Community-level variance with standard deviation, intracluster correlation coefficient (ICC), Proportional Change in Community Variance (PCV), and median odds ratio (MOR) were used as indicators of heterogeneity (random-effects). The median odds ratio (MOR) is used to transform area level variance into the commonly used odds ratio (OR) scale, which has a consistent and intuitive interpretation. When randomly selecting two areas, the MOR is defined as the median value of the odds ratio between the area at the highest risk and the area at the lowest risk. The MOR can be conceptualized as the increased risk that (in median) would have if moving to another area with a higher risk. It is determined by $MOR={e}^{\sqrt{(2\times VA)}\times 0.6745}$ [31]. Where; VA is the variance of the region standard, and 0.6745 is the 75th percentile of the normal distribution's cumulative distribution function with mean 0 and variance 1, see the detailed definition [28]. Whereas the proportional variance shift is determined as [29] $PCV=[(VA-VB)/VA]*100\%$, where; VA = original model variance and VB = model variance with more terms.

## Result

### Socio-demographic characteristics of study participants in Sub-Saharan Africa

A total of 328, 789 children who was born in the last five years preceding each country’s DHS survey were included in this study.

Majority of women 138,614 (42.15%) have no education while 107,871 (32.80%) have been primary education in the Sub-Saharan Africa. The large number of women were married 235,871 (71.72%). The majority of women were delivered at health facility 208,554 (63.42%) and the large number of women were attend ANC visit at the second trimester 116,773 (60.05%) (Table 1). More than half of 84,440 (55%) of women do not have immediate skin-to-skin contact in the area.

**Table 1:** Socio-demographic characteristics of study participants in Sub-Saharan Africa

Variables	Ferequency	Percentage
**Women education**		
No education	138,614	42.15
Primary	107,871	32.80
Secondary	82,372	25.05
**Weaklth Index**		
Poor	158,196	48.10
Middle	65,092	19.79
Rich	105,569	32.10
**Womens age**		
< 20	197,416	60.94
20–34	125,677	38.80
34 +	840	0.26
**Marital status**		
Single	20,295	6.17
Married	235,871	71.72
Divorced	72,691	22.10
**Huasband/partner educational status**		
No education	111,090	39.38
Primary	76,266	27.04
Secondary	94,734	33.58
**Birth order of child**		
1	70,759	21.52
2–3	113,740	34.59
4–5	75,051	22.82
6 +	69,307	21.08
**Trimester at ANC visit**		
1^st^ trimester	73,876	37.99
2^nd^ trimester	116,773	60.05
3^rd^ trimester	3,822	1.97
**No of ANC vist**		
None	24,983	11.22
1–3	76,592	34.40
4 +	121,076	54.38
**Place of delivery**		
Home	120,303	36.58
Health Institution	208,554	63.42
**Residence**		
Rural	231,860	70.52
Urban	96,929	29.48
**Size of child at birth**		
Large	110,170	35.22
Average	149,801	47.89
Small	52,836	16.89
**Skin to skin contcat**		
Yes	69,230	45.05
No	84,440	54.95
**Media exposure**		
Yes	203,785	62.08
No	124,460	37.92
**Mode of delivery**		
Vaginal	309,345	95.70
Cesearian section	13,898	4.30
**Birth Interval**		
≤ 24 months	58,637	17.83
> 24 months	270,152	82.17
**Sex of child**		
Male	166,539	50.65
Female	162,250	49.35
**Parity**		
1	45,807	13.93
2–3	119,019	36.19
4–5	82,230	25.00
6 +	81,801	24.87

*ANC* Antenatal care visit

### Pooled prevalence of early initiation of feeding in Sub-Saharan Africa

**Fig.1:**
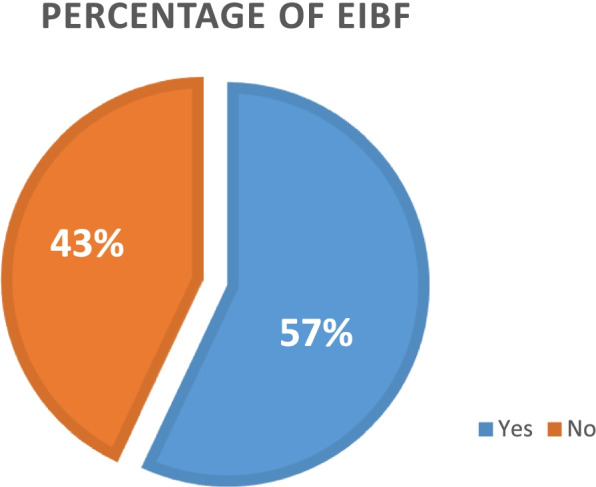
Pooled prevalence of early initiation of breastfeeding in Sub-Saharan Africa from 2010–2020 DHS data set

The pooled prevalence of early initiation of breastfeeding in Sub-Saharan African countries was 57% (Fig. 1) with 95% confidence interval (55.84 – 60.51%) with the highest early initiation of breastfeeding was found in Burundi (86%) while the lowest percentage of early initiation of breastfeeding was practiced in Congo Brazzaville (24%) (Table 2).

**Table 2:** The demographic and health survey characteristics of children in Sub-Saharan Africa

Country	Survey year	Number study participants (n)	Percentage EIBF
Angola	2015–16	11,572	48.34
Burkina Faso	2010	14,662	43.26
Benin	2017–18	12,159	55.15
Burundi	2016–17	12,111	85.86
CDR	2011	17,780	51.78
Congo	2013	8,200	23.58
Cote d’ Ivoire	2011	7,258	27.21
Cameron	2018	8,999	54.20
Ethiopia	2016	10,053	73.12
Gabon	2012	4,146	38.42
Ghana	2014	5,698	55.62
Gambia	2013	7,471	53.57
Guinea	2018	7,453	45.09
Kenya	2013	20,508	66.35
Comoros	2012	2,218	36.74
Liberia	2013	7,091	59.68
Lesotho	2014	2,569	70.54
Mali	2018	8,795	66.54
Malawi	2015–16	16,400	81.00
Niger	2012	11,460	56.63
Nigeria	2018	4,051	74.47
Namibia	2013	7,679	79.62
Ruanda	2014–15	8,509	78.61
Serra Leone	2019	10,964	51.92
Senegal	2010–11	17,426	27.85
Chad	2014	6,683	59.04
Togo	2013–14	9,129	53.34
Tanzania	2014–15	14,409	67.76
Uganda	2016	3,291	75.63
South Africa	2016	8,841	78.57
Zambia	2018	4,267	58.19
Zimbabwe	2015	32,013	43.26
All		328,789	100%

*CDR* Congo democratic republic

### Determinants of EIBF in Sub-Saharan Africa

#### Random effect analysis result

The fixed effects (a measure of association) and the random intercept for early initiation of breastfeeding are presented in Table 3. The result of the empty model revealed that there was significant variablity in the odds of practicing early initiation of breastfeeding with community variance ($\tau =0.83, p<0.001$). In addition, the MOR was 2.38(95% CI 2.19 – 2.60) meaning that the odds of practicing early initiation of breastfeeding were 2.38 times higher when respondents moved from low to high risk communities. This revealed that the existence of significant heterogienity in providing early initiation of breastfeeding across different communities. In the full model (model adjusted for both individual and community level factors) community variance (community variance = 0.52; *p*-value < 0.001) remained significnt but reduced. About 52% of the total variation of practicing early initiation of breastfeeding can be attributed to the contextual level factors that remained sign ficant even after considering some contextual risk factors. The proportional change in variance (PCV) in this model was 37.35% which indicates that 37.35% of community variance observed in the null model was explained by both the community and individual level variables (Table 3).

**Table 3:** Community level variability and model fitness for assessment of early initiation of breastfeeding among women of reproductive age in Sub-Saharan Africa

Parameter	Null model	Model I	Model II	Model III
Community variance(se)	0.83(0.043)	0.74(0.074)	0.66(0.042)	0.52(0.09)
ICC	0.19	0.16	0.18	0.16
MOR(95% CI)	2.38(2.19 – 2.60)	2.27(1.96 – 2.63)	2.17(1.95 – 2.30)	2.00(1.67 – 2.37)
PCV(%)	Reference	10.84%	20.48%	37.35%
Model comparision				
Log-liklihood ratio	-138,905.70	-56,638.26	-138,895.90	-56,636.35
Deviance (-2LL)	277, 811.4	113,276.52	277, 791	113, 272.7

-*2LL* log-likelihood, *ICC* Intra class Correlation Coefficient, *MOR* Median Odds Ratio, *PCV* Proportional Change in Variance, *SE* Standard Error

#### The fixed effect analysis result

The model with smaller deviance and the largest likelihood (MODEL IV) was best fit the data and the interpretation of the fixed effects was based on this model. Model IV was adjusted for both individual and community – level factors. Consequently, house hold wealth quantile, birth order, women educational status, place of delivery, health insurance, size of child at birth, immediate skin-to-skin contact, mode of delivery, media exposure, and husband educational status are significantly associated with early initiation of breastfeeding in Sub-Saharan Africa. The odds of practicing early initiation of breastfeeding among rich was 1.23 times higher than that of poor (AOR 1.23; 95% CI = 1.23—1.34).

The likelihood of practicing early initiation of feeding were 1.20 times higher among women who have primary education as compared to none-educated women (AOR = 1.20; 95% CI = 1.16—1.26). The odds of providing early initiation of breastfeeding among women who delivered at health facility were 2.00 times higher as compared to home delivery (AOR = 2.00; 95% CI = 1.89—2.05). The odds of providing early initiation breastfeeding among women whose size of child was average were 7% times higher as compared to large birth size while the odds of providing early breastfeeding among small birth size were 13% times lower as compared large birth size (AOR = 1.07; 95% CI = 1.03—1.11) and (AOR = 0.87; 95% CI = 0.83—0.92) respectively.

The odds of practicing early initiation of breastfeeding among women who has immediate skin to skin contact to their newborn child were 51% higher as compared to those who did not immediate skin to skin contact (AOR = 1.51; 95% CI = 1.47—1.57). The likelihood of practicing early initiation of breastfeeding were 73% times lower among women delivered by caesarean section as compared to vaginal delivery (AOR = 0.27; 95% CI = 0.25 – 0.29). The odds of providing EIBF among individuals who has been health insurance coverage were 53% higher as compared to individuals who have not health insurance (AOR = 1.53; 95% CI = 1.42 – 1.65). Also, the odds of practicing early initiation of breastfeeding were increased by 36% among women who have media exposure in Sub-Saharan Africa (AOR = 1.36; 95% CI = 1.31 – 1.41) (Table 4).

**Table 4:** A multilevel multilvariable analysis of factors associated with early initiation of breastfeeding in Sub-Saharan Africa from 2010–2020

Variables	Model II AOR (95% CI)	Model III	Model IV AOR (95% CI)
**Women education**			
No education	1.00		1.00
Primary	1.22(1.15—1.27)		1.20 (1.16—1.26)^a^
Secondary +	1.03(0.98—1.09)		1.06(1.01—1.12)^b^
**Weaklth Index**			
Poor	1.00		1.00
Middle	1.16(1.12—1.21)		1.10 (1.06 1.16)^a^
Rich	1.28(1.23—1.35)		1.29 (1.23—1.34)^a^
**Womens age**			
< 20	1.00		1.00
20–34	1.04 (1.01—1.08)		1.04(1.01—1.09)^b^
34 +	0.79 (0.61—1.04)		0.80 (0.61—1.04)
**Huasband/partner education**			
No education	1.00		1.00
Primary	1.12 (1.07—1.17)		1.11 (1.06—1.16)^a^
Secondary +	0.88 (0.84—0.93)		0.87 (0.83—0.92)^a^
**Birth order of child**			
1	1.00		1.00
2–3	1.27 (1.22—1.33)		1.27(1.22—1.33)^a^
4–5	1.25 (1.19—1.31)		1.25(1.19—1.32)^b^
6 +	1.16 (1.11—1.23)		1.17(1.11—1.23)^b^
**Trimester at ANC visit**			
1^st^ trimester	1.00		1.00
2^nd^ trimester	0.96 (0.93—1.00)		0.96 (0.93—1.01)
3^rd^ trimester	0.94 (0.84—1.05)		0.94 (0.84—1.05)
**ANC vist**			
No	1.00		1.00
Yes	1.01 (0.97—1.07)		1.01 (0.97—1.05)
**Place of delivery**			
Home	1.00		1.00
Health Institution	1.97(1.89—2.05)		1.97(1.89—2.05)^a^
**Health Insurance**			
No	1.00		1.00
Yes	1.53 (1.43—1.65)		1.53(1.42—1.65)^a^
**Size of child at birth**			
Large	1.00		1.00
Average	1.07(1.03—1.11)		1.07(1.03—1.11)^a^
Small	0.87(0.84—0.92)		0.87(0.83—0.92)^a^
**Skin to skin contcat**			
No	1.00		1.00
Yes	1.51 (1.47—1.57)		1.51(1.47—1.57)^a^
**Media exposure**			
No	1.00		1.00
Yes	1.31 (1.30 – 1.40)		1.36 (1.31—1.41)^a^
**Mode of delivery**			
Vaginal	1.00		1.00
Cesearian section	0.27 (0.25—0.29)		0.27 (0.25—0.29)^a^
**Sex of child**			
Male	1.00		1.00
Female	1.02 (0.99—1.05)		1.02 (0.99- 1.05)
**Birth Interval**			
< 24	1.00		1.00
≥ 24	1.00(0.95—1.04)		1.00 (0.96—1.04)
**Residence**			
Rural		1.00	1.00
Urban		1.00(0.98—1.02)	1.30 (1.26—1.36)^a^
**Community poverty**			
Low		1.00	1.00
High		0.96(0.93—1.04)	1.02 (0.96—1.07)
**Community ANC visit**			
Low		1.00	1.00
High		1.02(0.98—1.07)	1.00 (0.94—1.06)
**Community education**			
Low		1.00	1.00
High		1.06 (1.02—1.06)	1.00(0.94—1.06)
**Community media exposure**			
Low		1.00	1.00
High		0.99 (0.96—1.03)	1.00 (0.94—1.05)

AOR = adjusted odds ratio

^a^significant at 0.01

^b^significant at 0.05

*signficant at 0.1

## Discussion

The overall objective of this study was to investigate the pooled prevalence and determinants of early initiation of breastfeeding practice among mothers who have children less than 5 years in Sub-Saharan Africa from 2010–2019 using recent Demographic and Health data set. In this study we found that the pooled prevalence of EIBF in Sub-Saharan Africa was 57% this is lower than WHO and UNICEF recommendation [3, 32]. This could be due to limited awareness and perceptions regarding the relevance of early initiation of breastfeeding and colostrum to their newborns health over his lifetime, hence delayed initiation of breastfeeding and prelacteal feeding still remains a public health concerns in those regions. Community and facility awareness and education programs are also required, as WHO and UNICEF suggest that every newborn be placed on the mother's breast within the first hour of life because it "gives them the best chance to survive" [33, 34]. The highest prevalence of EIBF found in Burundi and Malawi while the lowest prevalence was reported in republic of Congo. The divergence of the EIBF outcome may be different socio-cultural difference and discrepancy on the implementation of international infant feeding practice on health care workers as well as on the government side.

Covariates significantly correlated with early initiation of breastfeeding in Sub-Saharan Africa was mode of delivery, women educational status, skin-to-skin contact, Husband educational status, household wealth index, health insurance, media exposure and institutional delivery.

In this study mode of delivery was strongly correlated with early initiation of breastfeeding, hence women with caesarean delivery were 83% less likely to practice early initiation of breastfeeding compared to vaginal delivery. Similar outcomes are observed in other studies conducted in Tanzania, South Asia, Zimbabwe, and Ethiopia [7, 18, 23, 35–39], since women who have undergone caesarean section may have endured prolonged recovery from anaesthesia pain, fear and stress that leads to delay the flow of milk in the breast as well as infants born by caesarean section are more likely to have respiratory distress that could cause a newborn to be taken to the intensive care unit resulted in separating from mother. Place of delivery is a strong predictors of early initiation of breastfeeding in this study, the odds of practicing EIBF almost 2 times higher among women who deliver in health facility as compared to home delivery. Since a women delivered in health facility have been pressured by health care workers to provide breast milk to their newborns early, similar outcomes are published in other studies [27, 35, 37, 40].

In this study we found that maternal education significantly correlated with early initiation of breastfeeding in line with other studies [10, 36, 41–43]. Hence educated women have the ability to understand the benefits of early initiation of breastfeeding and provision of colostrum to their newborns provided by health care workers as well as have better accessibility to attend ANC and have better media exposure. Also, early breastfeeding initiation is significantly correlated with birth order. This may be because previous experience with breastfeeding has a positive impact on the desire to practice timely initiation of breastfeeding, as well as previous experience with breastfeeding has a positive impact on improvements in women's beliefs about the practice of timely initiation of breastfeeding [40, 44, 45]. In addition, a women with middle and rich wealth quantile more likely to practice early initiation of breastfeeding consistent with other studies conducted in elsewhere [5, 46, 47]. This may be because women with greater financial access are able to obtain basic health care services during pregnancy and are able to pay for the services they receive in the health facility as well as transportation. Moreover, the likelihood of practicing early initiation of breastfeeding was 1.51 times higher among women who have immediate skin-to-skin contact as compared to delayed skin contact consistent with other studies conducted in Tanzania, Nigeria and Australia [27, 39, 48]. Hence skin-to-skin contact with the mother facilitates early breastfeeding by releasing the hormones prolactin, which stimulates lactation, and oxytocin, and encourages attachment to the mother. As a result, the WHO and UNICEF endorse the practice as part of the immediate newborn care package because it creates an optimal environment for breastfeeding the infant [3, 32]. Similarly the likelihood of early initiation of breastfeeding was 36% higher among women who have media exposure as compared to no media exposure in agreement with a studies conducted in Ghana, Jordan and Ethiopia [49–51]. Hence media is one of the important ways to promote the community regarding the benefits of early initiation of breastfeeding to their newborn health and survival in their lifetime as well as easily ways to address large number of communities in the specific country. The likelihood of practicing early initiation of breastfeeding 53% higher among women who has health insurance coverage as compared to without health insurance coverage. This could be the fact that those women who have health insurance coverage have the confidence to receive health care access in the area and able to reduce stress related to treatment during pregnancy.

### Strengths and limitation of the study

Regarding strengths, the data used in this study was obtained from nationally representative and the covariates in the 32 Sub-Saharan Africa DHS dataset were the same as well as comparable across all countries. The study was population based with a response rate of > 90% and the data were pooled together to create large sample size that upsurges the generalizability EIBF reported within 5 years preceding each country survey rages from 2010 to 2020. Also, the study was have the ability to identify the significant determinants of EIBF across 32 Sub-Sahara African countries to inform program planners and nutrition policy makers for prioritization and specific interventions. In case of limitation, the finding of this study may not establish a true causal relationship between the outcome variable due to the cross-sectional nature of the study design. The data was collected based on self-report from mothers within 5 years preceding the survey and this could be a potential recall bias.

## Conclusion

This research adds to our understanding of breastfeeding initiation practices in Sub-Saharan African countries with high levels of poverty. The magnitude of early initiation of breastfeeding rate in Sub-Saharan Africa was low, with a variation of EIBF between countries since the highest prevalence of EIBF was found in Malawi while the lowest EIBF was practiced in Congo Brazzaville. Media exposure, maternal educational status, place of delivery, mode of delivery, Health insurance coverage, and skin-to-skin contact were factors significantly associated with EIBF in this study. With caesarean delivery becoming more popular, it's vital that these women obtain additional breastfeeding support after delivery. Immediate skin-to-skin contact after delivery should be promoted and supported by health care staff. The government, as well as all other concerned bodies operating in the field of nutrition and child development, should put a greater focus on media accessibility for all populations. Furthermore, encouraging institutional delivery and raising awareness about the benefits of breastfeeding was strongly advised.

## Data Availability

Data is available online and you can access it from www.measuredhs.com.

## Abbreviations

ANC: Antenatal Care
AOR: Adjusted Odds Ratio
CI: Confidence Interval;
DHS: Demographic Health Survey
ICC: Intra-class Correlation Coefficient;
LLR: Log-likelihood Ratio
LR: Likelihood Ratio
MOR: Median Odds Ratio
SSA: Sub-Saharan Africa
WHO: World Health Organization

## References

[1] Victora CG (2016). Breastfeeding in the 21st century: epidemiology, mechanisms, and lifelong effect. The Lancet.

[2] Yenit MK, Genetu H, Tariku A (2017). Infant feeding counseling and knowledge are the key determinants of prelacteal feeding among HIV exposed infants attending public hospitals in Ethiopia. Archives of Public Health.

[3] Organization, W.H., Guideline: protecting, promoting and supporting breastfeeding in facilities providing maternity and newborn services. World Health Organization; 2017. 29565522

[4] Organization, W.H. (2009). Baby-friendly hospital initiative: revised, updated and expanded for integrated care.

[5] Smith ER (2017). Delayed breastfeeding initiation and infant survival: A systematic review and meta-analysis. PLoS One.

[6] Edmond KM (2006). Delayed breastfeeding initiation increases risk of neonatal mortality. Pediatrics.

[7] Nair N (2010). Improving newborn survival in low-income countries: community-based approaches and lessons from South Asia. PLoS Med.

[8] Aborigo RA (2012). Infant nutrition in the first seven days of life in rural northern Ghana. BMC Pregnancy Childbirth.

[9] Victoria C (2000). Effect of breastfeeding on infant and child mortality due to infectious diseases in less developed countries: a pooled analysis. Lancet (British edition).

[10] Acharya D (2018). Correlates of the timely initiation of complementary feeding among children aged 6–23 months in Rupandehi District. Nepal Children.

[11] Disha A (2015). Factors associated with infant and young child feeding practices in Amhara region and nationally in Ethiopia: analysis of the 2005 and 2011 demographic and health surveys.

[12] Karimi FZ (2019). The effect of mother-infant skin to skin contact on success and duration of first breastfeeding: A systematic review and meta-analysis. Taiwan J Obstet Gynecol.

[13] Balogun OO (2016). Interventions for promoting the initiation of breastfeeding. Cochrane Database Syst Rev.

[14] Dewey KG, Vitta BS (2013). Strategies for ensuring adequate nutrient intake for infants and young children during the period of complementary feeding.

[15] Ogbo FA (2017). Infant feeding practices and diarrhoea in sub-Saharan African countries with high diarrhoea mortality. PloS one.

[16] Workineh Y, Gultie T (2019). Latency period and early initiation of breastfeeding in term premature rupture of membrane in Southern Ethiopia, 2017. Ital J Pediatr.

[17] Tamiru D (2012). Sub-optimal breastfeeding of infants during the first six months and associated factors in rural communities of Jimma Arjo Woreda, Southwest Ethiopia. BMC Public Health.

[18] Mukora-Mutseyekwa F (2019). Predictors of early initiation of breastfeeding among Zimbabwean women: secondary analysis of ZDHS 2015. Maternal Health, Neonatology and Perinatology.

[19] MaJra J.P., Silan V.K. (2016). Barriers to early initiation and continuation of breastfeeding in a tertiary care Institute of Haryana: a qualitative study in nursing care providers. J Clin Diagnostic Res: JCDR.

[20] Temesgen H (2018). Prelacteal feeding and associated factors in Ethiopia: systematic review and meta-analysis. Int Breastfeed J.

[21] Vieira TO (2010). Determinants of breastfeeding initiation within the first hour of life in a Brazilian population: cross-sectional study. BMC Public Health.

[22] Legesse M (2014). Prelacteal feeding practices and associated factors among mothers of children aged less than 24 months in Raya Kobo district, North Eastern Ethiopia: a cross-sectional study. Int Breastfeed J.

[23] Smith ER (2017). Delayed breastfeeding initiation and infant survival: A systematic review and metaanalysis. PLoS ONE.

[24] Khan GN (2017). Determinants of infant and young child feeding practices by mothers in two rural districts of Sindh, Pakistan: a cross-sectional survey. Int Breastfeed J.

[25] Bhandari S (2019). Determinants of infant breastfeeding practices in Nepal: a national study. Int Breastfeed J.

[26] Sharma IK, Byrne A (2016). Early initiation of breastfeeding: a systematic literature review of factors and barriers in South Asia. Int Breastfeed J.

[27] Lyellu HY (2020). Prevalence and factors associated with early initiation of breastfeeding among women in Moshi municipal, northern Tanzania. BMC Pregnancy Childbirth.

[28] Demographic T, Program HS*.* Guide to DHS Statistics.

[29] Goldstein, H., Multilevel statistical models. Vol. 922. John Wiley & Sons; 2011.

[30] Diez-Roux AV (2000). Multilevel analysis in public health research. Annu Rev Public Health.

[31] Goldstein H, Browne W, Rasbash J (2002). Partitioning variation in multilevel models. Understanding statistics: statistical issues in psychology, education, and the social sciences.

[32] UNICEF W (2018). Capture the Moment–Early initiation of breastfeeding: The best start for every newborn.

[33] Abie BM, Goshu YA (2019). Early initiation of breastfeeding and colostrum feeding among mothers of children aged less than 24 months in Debre Tabor, northwest Ethiopia: a cross-sectional study. BMC Res Notes.

[34] WHO, U (2014). Every newborn: an action plan to end preventable deaths.

[35] Karim F (2018). Initiation of breastfeeding within one hour of birth and its determinants among normal vaginal deliveries at primary and secondary health facilities in Bangladesh: a case-observation study. PloS one.

[36] Adhikari M (2014). Factors associated with early initiation of breastfeeding among Nepalese mothers: further analysis of Nepal Demographic and Health Survey, 2011. Int Breastfeed J.

[37] Exavery A (2015). Determinants of early initiation of breastfeeding in rural Tanzania. Int Breastfeed J.

[38] Belachew A (2019). Timely initiation of breastfeeding and associated factors among mothers of infants age 0–6 months old in Bahir Dar City, Northwest, Ethiopia, 2017: a community based cross-sectional study. Int Breastfeed J.

[39] Arora A (2017). Determinants of breastfeeding initiation among mothers in Sydney, Australia: findings from a birth cohort study. Int Breastfeed J.

[40] Derso T (2017). Correlates of early neonatal feeding practice in Dabat HDSS site, northwest Ethiopia. Int Breastfeed J.

[41] Kiwango F (2020). Prevalence and factors associated with timely initiation of breastfeeding in Kilimanjaro region, northern Tanzania: a cross-sectional study. BMC Pregnancy Childbirth.

[42] Ezeh OK (2019). Factors Associated with the Early Initiation of Breastfeeding in Economic Community of West African States (ECOWAS). Nutrients.

[43] Ahmed AE, Salih OA (2019). Determinants of the early initiation of breastfeeding in the Kingdom of Saudi Arabia. Int Breastfeed J.

[44] John JR (2019). Determinants of early initiation of breastfeeding in Ethiopia: a population-based study using the 2016 demographic and health survey data. BMC Pregnancy Childbirth.

[45] Mukunya D (2017). Factors associated with delayed initiation of breastfeeding: a survey in northern Uganda. Glob Health Action.

[46] Tilahun G (2016). Prevalence and associated factors of timely initiation of breastfeeding among mothers at Debre Berhan town, Ethiopia: a cross-sectional study. Int Breastfeed J.

[47] Horii N (2017). Determinants of early initiation of breastfeeding in rural Niger: cross-sectional study of community based child healthcare promotion. Int Breastfeed J.

[48] Shobo OG (2020). Factors influencing the early initiation of breast feeding in public primary healthcare facilities in Northeast Nigeria: a mixed-method study. BMJ open.

[49] Seidu A-A (2020). Determinants of early initiation of breastfeeding in Ghana: a population-based cross-sectional study using the 2014 Demographic and Health Survey data. BMC Pregnancy Childbirth.

[50] McDivitt JA (1993). The impact of the Healthcom mass media campaign on timely initiation of breastfeeding in Jordan. Stud Fam Plann..

[51] Bimerew A, Teshome M, Kassa GM (2016). Prevalence of timely breastfeeding initiation and associated factors in Dembecha district, North West Ethiopia: a cross-sectional study. Int Breastfeed J.

